# Reactivity of the Oxytocinergic and Neuroendocrine System Following the Adult Attachment Projective Picture System in Men of Recent Fatherhood: Results from an Exploratory Pilot Study with a Cross-Sectional Design

**DOI:** 10.3390/brainsci12101314

**Published:** 2022-09-28

**Authors:** Alexander Karabatsiakis, Karin de Punder, Cornelia Doyen-Waldecker, Laura Ramo-Fernández, Sabrina Krause, Anja Maria Gumpp, Alexandra Maria Bach, Jörg Michael Fegert, Iris-Tatjana Kolassa, Harald Gündel, Ute Ziegenhain, Anna Buchheim

**Affiliations:** 1 Department of Clinical Psychology II, Institute of Psychology, University of Innsbruck, 6020 Innsbruck, Austria; 2 Department of Psychosomatic Medicine and Psychotherapy, Ulm University, 89081 Ulm, Germany; 3 Institute of Psychology and Education, Clinical & Biological Psychology, Ulm University, 89081 Ulm, Germany; 4 Department of Child and Adolescent Psychiatry and Psychotherapy, Ulm University Hospital, 89081 Ulm, Germany

**Keywords:** stress, attachment representation (AR), Adult Attachment Projective Picture System (AAP), early fatherhood, saliva, biomarkers, oxytocin, cortisol, dehydroepiandrosterone (DHEA)

## Abstract

The attachment representation (AR) of individuals affects emotional and cognitive information processes and is considered an important modulating factor of neuroendocrine stress responses. The neuropeptide oxytocin is studied as one biomolecular component underpinning this modulation. A validated procedure used in attachment-related research is the Adult Attachment Projective Picture System (AAP). To date, only a limited number of studies investigated oxytocin and neuroendocrine reactivity in the context of an attachment-related stimulus similar to the APP. In this pilot study, N = 26 men of recent fatherhood were exposed to the AAP to classify AR and to investigate salivary changes in oxytocin, cortisol and dehydroepiandrosterone (DHEA) after AAP stimulation. We observed increased oxytocin levels in response to AAP exposure, and this increase was more pronounced in fathers with unresolved/disorganized AR. No significant changes in cortisol and DHEA concentrations were observed in response to AAP administration. Interestingly, differences in basal cortisol levels (before the AAP) also depended on AR classification. Here, the group of men with unresolved/disorganized AR showed higher levels of cortisol compared to fathers with organized AR. To conclude, the finding of increased salivary oxytocin levels in response to the AAP further indicates its validity as an instrument to stimulate the attachment system.

## 1. Introduction

The ability to form attachment is developed and shaped in early childhood and is indispensable for human social relationships [1]. According to the attachment theory, attachment is an inborn psychobiological system that stimulates humans to search for psychological and physical proximity to their caregivers when in need of security [1]. Research indicates that the character or quality of one’s childhood attachments with primary caregivers shapes an individual’s attachment representation (AR). In addition, it affects emotional and cognitive information processes involved in attention and memory as well as the emotional and neuroendocrine (stress) reactivity of the central nervous system [1,2,3,4,5,6]. AR seems to be an important determinant in clinical psychology and psychiatry, as attachment insecurity contributes to a wide spectrum of stress-associated mental disorders [5,7]. Therefore, an improved understanding of the (biomolecular) mechanisms underlying the association between AR and psychiatric disease risk is of key importance for the development of improved clinical interventions based on improved therapeutic strategies.

The Adult Attachment Projective Picture System (AAP) is a valid indicator of attachment status [8,9], designed to activate an individual’s internal working model of attachment by introducing attachment-related topics (e.g., separation, illness, abuse and death). Using the AAP, attachment classifications are based on the analysis of narrative responses to a standardized set of eight projective pictures and include three organized/resolved AR, namely secure, dismissing, preoccupied and an unresolved/disorganized AR related to experiences of trauma or loss [2,6,10]. The narrative of individuals who are unresolved/disorganized demonstrates their inability to contain or integrate fearful thematic elements. Moreover, during the AAP, these individuals are unable to use defensive processes to remain organized and recover from conscious thoughts and feelings of fearful distress. Prior research indicated that the AAP can be applied as an attachment-related stimulus to activate neurobiological pathways involved in attachment, including the oxytocinergic system [6,10,11].

Psychobiological stress research identified the neuropeptide oxytocin as a promising biomarker candidate for investigating the biomolecular underpinnings of attachment and psychosocial behaviors. In the brain, oxytocin is mainly produced in the paraventricular nucleus (PVN) of the hypothalamus and released into the systemic circulation by the posterior pituitary gland, initiating a wide range of biological effects. In addition, oxytocin exerts its effects in the central nervous system through a slow, “unwired” and global transmission of OT that is released mainly from neuronal dendrites [12] and via “hard-wired” oxytocinergic nerve fibers from the hypothalamus to many other brain regions, including the medial prefrontal cortex, amygdala, hippocampus, nucleus accumbens and ventral tegmentum [13,14]. Through these pathways, oxytocin is a physiological regulator of social behavior in mammals (for a review, see [15]) and plays a key role in maternal caregiving behavior, attachment security, partner binding and interpersonal trust [16,17,18].

Oxytocin not only regulates social behavior but is also a modulator of the physiological stress response mediated by the hypothalamic-pituitary-adrenocortical (HPA)-axis [19,20,21]. Central signaling molecules of HPA-axis regulation include the steroid hormone cortisol and its co-regulator dehydroepiandrosterone (DHEA) [22,23]. Pioneering work demonstrated a stress-buffering effect of intranasal oxytocin administration in combination with social support on salivary cortisol levels using an acute laboratory stressor (Trier Social Stress test, TSST) [24]. These results were replicated several times, and a meta-analysis examining the acute effects of intranasal oxytocin administration on the cortisol stress response indicated that buffering effects of oxytocin on the stress response were most pronounced in laboratory settings that produce robust HPA-axis responses [25].

Endogenous levels of oxytocin have been repeatedly found to influence the neuroendocrine system and inhibit the release of adrenocorticotropic hormone (ACTH) and cortisol in both animal and human studies [19,20,21]. Although the literature is not conclusive on whether peripheral oxytocin levels change after acute stress exposure in humans [26], several studies observed oxytocin and cortisol co-activation in response to a laboratory stressor [26,27,28,29]. It has been suggested that the increase in oxytocin concentration, which peaks before cortisol reaches its maximum, has stress-reducing effects by boosting the recovery of HPA-axis activities [26]. One study investigated the interplay of adult attachment, oxytocin and the neuroendocrine stress system in the context of the TSST. The highest plasma oxytocin levels were observed in individuals with secure AR, and in addition, these individuals displayed moderate HPA-axis responses and relatively low levels of subjective stress, while subjects with an unresolved/disorganized AR showed higher levels of subjective stress and a blunted HPA-axis response [4]. Studies performed in animals and humans suggest that limbic structures that are involved in stress processing and attachment development, such as the amygdala and hippocampus, are affected by the attachment status [6,30,31], possibly explaining the observed differences in oxytocin HPA-axis responses.

Most studies to date investigated the interplay between oxytocin and the neuroendocrine stress response in the context of an acute psychosocial stressor such as the TSST. However, little is known about the effect of attachment-related stimuli on oxytocin and neuroendocrine reactivities. To the best of our knowledge, only three studies investigated oxytocin and/or cortisol reactivity in response to an attachment-related stimulus [3,11,32]. For example, elevations in cortisol in response to the Adult Attachment Interview (AAI) correlated with dismissive AR in patients with a diagnosis of idiopathic spasmodic torticollis (IST) [3]. Krause et al. [11] demonstrated elevated oxytocin and decreased cortisol concentrations in serum after AAP exposure compared to baseline in women three months after parturition. Finally, higher baseline oxytocin levels but no differences in oxytocin reactivity were observed in patients with polydrug user disorder compared to healthy controls following the administration of the AAP [32].

To gain further insights into the biological effects of AAP exposure, here, we investigated basal and reactivity measures of selected attachment- and stress-related biomarkers (oxytocin, cortisol and DHEA) in a laboratory setting in men who recently became fathers. We hypothesized that (i) exposure to AAP as an attachment stimulus is associated with a salivary increase in oxytocin and DHEA together with a decrease in cortisol levels and (ii) changes in hormone levels are associated with the attachment classification of the individual and that (iii) attachment classifications are associated with differences in basal levels of oxytocin, cortisol and DHEA in saliva.

## 2. Materials and Methods

### 2.1. Recruitment and Study Design

This work was conducted as a side project and a pilot study related to the central project “My childhood–Your childhood” funded by the German Federal Ministry of Education and Research (BMBF). The central project focused on the identification of risk and resilience factors in the inter-generational transmission of childhood maltreatment [33]. In this side project, the study cohort was extended by the recruitment and inclusion of men who recently became fathers. Both studies fulfilled the guidelines of the Declaration of Helsinki [34] and were approved by the Ethics Committee of Ulm University. Men of recent fatherhood were recruited via public advertisements as well as within the maternity ward of the Ulm University Hospital.

A total of *n* = 134 mothers from the main study were informed about the father project and were asked to forward the information letter about the project and the contact form to the father of the child. Unfortunately, direct contact with the father was not possible due to the obligation of confidentiality towards the mother participating in the main study. In addition, men of recent fatherhood (3 to 8 months postpartum) were recruited across 22 months via public advertisements as well as within the maternity ward of the Ulm University Hospital where the recruitment of women who recently became mothers was conducted for participating in central project TRANS-GEN.

In total, 26 men reported interest and agreed to participate. Inclusion criteria were as follows: a minimum of 18 years of age (adulthood at the time point of becoming a father), no reported current psychopathology or substance abuse and no current medication with known effects on the immune or neuroendocrine system. Exclusion criteria included insufficient knowledge of the German language. After passing the participation criteria and providing written informed consent, all N = 26 individuals were invited to a laboratory appointment starting around 9:30 a.m. The participants first provided basic demographic information by answering a battery of self-report questionnaires. The sociodemographic and clinical characteristics of the participants are presented in Table 1.

This quasi-experimental study used a pre-post intervention design with the AAP being the exposure or intervention. The outcome variables were salivary biomarker reactivity measures. In addition, both basal and reactivity measures were compared between two subgroups (fathers with organized vs. disorganized AR).

**Figure 1:**
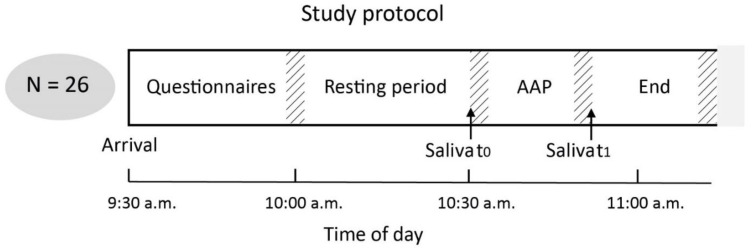
Graphical representation of the study protocol including the experimental sections, time intervals and time points (t_0_ = Before AAP, t_1_ = After AAP) of saliva collection. AAP, Adult Attachment Projective Picture System. Notification: Hatched areas indicate inter-individual differences for the duration of the AAP. The given time intervals represent the mean duration time (in min). For detailed information about grand averages and group-wise averages of duration times, also see Table 1.

After answering all questionnaires, a standardized time interval of 30 min was provided for relaxation before the first saliva sampling was conducted (time point t_0_, “Before AAP”), which took place shortly before the start of the AAP. After completing the AAP, a second saliva sample was collected (time point t_1_, “After AAP”). For a graphical representation of the experimental design and temporal duration, please see Figure 1.

### 2.2. Assessment of Attachment Representation Using the AAP

ARof the participants were determined using the AAP [35]. In general, the AAP consists of a set of eight pictures, representing one neutral and seven attachment-relevant scenes. Each picture was presented in a standardized order to the participants, which were asked to tell a short story related to each picture after receiving the following initial instruction (in German language): “What is going on in the picture, what led up to the scene, what are the characters thinking or feeling and what might happen next?”. Each picture stimulus was coded for content and defensive processes (for a more detailed description of the coding and classification procedure, see George and West [9,35]. The AAP is used to classify the four established attachment categories: Secure (F), preoccupied (E), dismissing (Ds) and unresolved (U). The AAP has demonstrated solid psychometric properties, including test-retest reliability, inter-rater reliability and convergent and discriminant validity [8,9,36]. The AAP classification in the present study was performed by two independent certified judges who agreed in 25 out of 26 cases. The one remaining case was judged in a consensus rating. See also Table 1 for the frequencies of the four different AR clusters in the study cohort. Considering the relatively small sample size of our pilot study and the resulting group sizes for the individual attachment classifications, we used the categorization “resolved/organized” (i.e., either “secure”, “insecure-dismissing”, “insecure-preoccupied”) vs. “unresolved/disorganized” AR in our analysis. Individuals with an organized attachment are able to contain (i.e., resolve) any frightening or threatening material (e.g., desperately alone, death, attack, abuse, helplessness, danger, failed protection and isolation) that may appear in the story. The unresolved attachment refers to individuals who are not able to regulate or reorganize stories containing frightening or threatening material and seem to be overwhelmed and dysregulated by their attachment fears. Differences in sociodemographics and clinical characteristics between fathers with an organized vs. disorganized/unresolved AR are listed in Table 1. Fathers with a disorganized/unresolved AR were older (t_24_ = −2.66, *p* = 0.014) and reported more cigarette smoking (χ^2^ = 7.9, *p* = 0.005) compared to fathers with an organized AR.

### 2.3. Sample Preparation

Saliva collection was performed by using sterile kits containing SalivaBio oral swabs and sampling tubes (Salimetrics, Carlsbad, CA, USA). After collection, saliva samples were immediately processed by centrifugation at 4 °C (pre-chilled centrifuge) and 1500× *g* for 15 min (Heraeus Megafuge 16R, Thermofisher Scientific, Waltham, MA, USA). From the total sample volume, one aliquot of 300 µL was used for the analysis of oxytocin, and two additional aliquots of 200 μL for cortisol and DHEA. All aliquots were handled on ice and immediately stored at −80 °C until further analyses. Oxytocin and cortisol measurements were prioritized when insufficient amounts of saliva were available (which was the case for *n* = 3 participants). The final sample size with valid DHEA measurement was *n* = 25 for t_0_ and *n* = 23 for t_1_. To avoid an artificial reduction in the available dataset for statistical analyses, all available biological measures were included in the analyses.

### 2.4. Quantification of Biomarkers in Saliva

Saliva aliquots for the measurement of oxytocin were shipped frozen on dry ice to the laboratory of Prof. Landgraf (RIAgnosis, Sinzing, Germany). Oxytocin concentrations were determined by a standardized radioimmune assay (RIA), as previously reported [11]. Cortisol and DHEA concentrations in saliva were assessed in duplicates using commercially available enzyme-linked immunosorbent assays (ELISA, Helsinki, Finland; IBL, Hamburg, Germany) according to the manufacturer’s protocols. Intra- and inter-assay coefficients of variability for the cortisol assay were 3.89 % and 4.1 %, respectively, and 12.54 % and 4.1 %, respectively for the DHEA assay.

### 2.5. Statistical Data Analyses

Statistical data analyses were performed using IBM SPSS Statistics (SPSS 26.0, Inc., Chicago, IL, USA), and figures were generated with version 3.2.3 of the statistical software R (R Core Team, 2015). First, the normal distribution of data was tested using Kolmogorov–Smirnov tests. Baseline differences (t_0_, before AAP exposure) in demographics and clinical characteristics and salivary biomarker measures between fathers with organized and disorganized AR were tested using the χ^2^-test (binary data), the independent Student’s *t*-test (normally distributed data) or the Mann–Whitney U-test (non-normally distributed data). Differences in salivary measures from before to after the AAP were tested using the paired Student’s *t*-test for normally distributed data (cortisol, DHEA) and the Wilcoxon test for non-normally distributed data (oxytocin t_1_, after AAP). Measures of reactivity were determined by calculating the percent change (([After the AAP] − [Before AAP]/ [Before the AAP]) * 100) and compared between “organized” and “unresolved/disorganized” fathers using Mann–Whitney U-tests for non-normally distributed data (oxytocin, cortisol and DHEA reactivity). Pearson’s (normally distributed data) and Kendall’s Tau (non-normally distributed data) correlations were applied to test for associations between basal and reactivity measures of oxytocin, cortisol and DHEA. For all analyses, a *p*-value < 0.05 was considered statistically significant. Because of the relatively small sample size of our pilot study, we could not fully control for the influence of potential confounders. However, we observed no significant correlations between the potential confounding variables age, BMI and cigarette smoking and baseline and reactivity biomarker variables (all *p*-values > 0.05), with an exception of the relationship between age and DHEA response (*p* = 0.009). We observed a trend towards a significant effect (*p* < 0.1) between age and oxytocin t_0_ and t_1_ levels.

## 3. Results

### 3.1. Reactivity Measures of Oxytocin, Cortisol and DHEA

**Figure 2:**
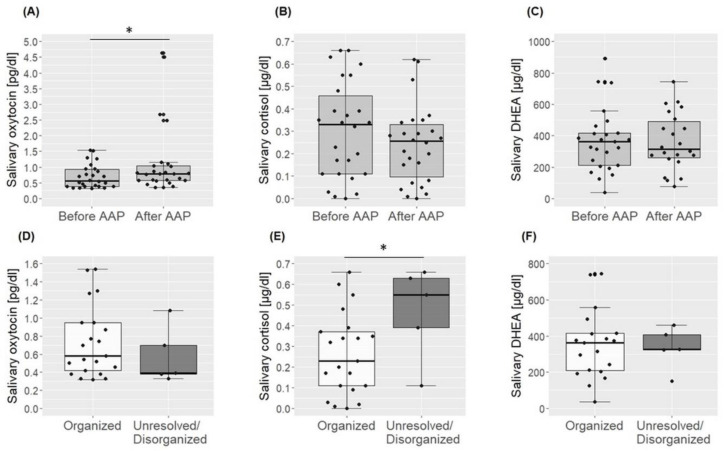
Graphical representation of results from the analysis of oxytocin, cortisol and DHEA before and after the AAP for the total cohort in light grey boxes (**A**–**C**) and before the AAP between fathers with an organized (white boxes) an unresolved/disorganized (dark grey boxes) AR (**D**–**F**). * *p* < 0.05.

On average, salivary oxytocin levels significantly increased in response to AAP exposure (*T* = 236, *z* = −1.98, *p* = 0.048), and this increase was more pronounced in unresolved/disorganized fathers compared to fathers with an organized AR (*U* = 84, *z* = 2.0, *p* = 0.041). Mean cortisol levels showed a trend towards a significant decrease at t_1_ (t_25_ = 1.93, *p* = 0.065), but no difference in cortisol reactivity between the different ARs was observed (*U* = 36, *z* = −1.1, *p* = 0.31). The mean DHEA concentration did not change in response to the AAP (t_22_ = −0.21, *p* = 0.84). Again, no differences in reactivity level between fathers with an unresolved/disorganized and organized AR were observed (*U* = 51, *z* = 0.45, *p* = 0.69). For an overview of t_0_, t_1_ and reactivity measures and a graphical representation of the results, see Table 1 and Figure 2A–C.

### 3.2. Basal Levels of Oxytocin, Cortisol and DHEA

Baseline levels of oxytocin (t_24_ = 0.82, *p* = 0.42) and DHEA (t_23_ = 0.42, *p* = 0.68) in fathers with an unresolved/disorganized AR did not differ significantly from the group with an organized AR. However, fathers with unresolved/disorganized AR showed higher baseline cortisol levels compared to fathers with an organized AR (t_24_ = −2.48, *p* = 0.021). See also Table 1 and Figure 2D–F for an overview of t_0_ baseline measures for fathers with organized and disorganized/unresolved AR.

### 3.3. Correlations among Salivary Biomarkers

Next, salivary biomarker data were tested for associations, first, separately among baseline and reactivity measures, but no significant results were found (data not shown, all *p*-values > 0.05). However, in response to AAP exposure, cortisol levels decreased more in individuals with higher baseline cortisol levels (*r* = −0.33, *p* = 0.018), while individuals with lower oxytocin baseline levels showed a stronger increase in oxytocin concentrations (*r* = −0.36, *p* = 0.011).

## 4. Discussion

Here, we examined oxytocinergic and neuroendocrine reactivities in response to the AAP in men who recently became fathers. Confirming our hypothesis, our findings suggest that AAP exposure induces the release of oxytocin, further advancing the use of the AAP as a procedure to stimulate the physiology of the attachment system. Contrary to our hypotheses, AAP administration did not induce changes in salivary cortisol and DHEA. We also observed that the oxytocin response differed by AR classification such that unresolved/disorganized fathers showed stronger increases in salivary oxytocin in response to the AAP. The group of unresolved/disorganized fathers, additionally, displayed higher baseline cortisol but showed no differences in oxytocin or DHEA levels compared to fathers with an organized AR. Taken together, the findings of this pilot study with fathers demonstrate that attachment status affects the functioning of both oxytocinergic and neuroendocrine systems.

Our results are consistent with previous findings in postpartum women showing increased plasma oxytocin levels in response to the AAP [11]. Although, in this study, no significant differences were observed between the different AR subgroups, mothers with an insecure AR tended to have stronger increases in oxytocin. Compared to healthy controls, males suffering from poly-drug-use disorder did not differ in oxytocin reactivity following AAP administration [32]. However, multi-drug users did not differ in AR from controls, making it rather difficult to directly compare these results with our study findings in AR subgroups. Other research investigating oxytocin levels before and after the TSST in association with AR showed that securely attached men displayed higher oxytocin levels over the course of the laboratory stressor compared to insecurely attached subjects [4]. Contrary, our findings indicated increased oxytocin reactivities in the group of insecure unresolved/disorganized men, but we administered an attachment- instead of a stress-related stimulus.

Here, we observed no significant differences in baseline oxytocin levels regarding AR. This result is inconsistent with previous findings, showing lower plasma oxytocin levels in women with an unresolved/disorganized AR compared to those with an organized AR [37]. An explanation for this discrepancy could be that this study was performed on women diagnosed with borderline personality disorder, while our study population is characterized by men who recently became fathers free of any physical or mental impairment. Another study investigating oxytocin levels in the context of attachment in young and older men and women found that lower plasma oxytocin levels were associated with higher attachment anxiety in young men [38]. In contrast, higher baseline blood oxytocin levels were observed in poly-drug users compared to healthy controls [32]. From a methodological point of view, the comparability between oxytocin levels in saliva and blood and their reactivity in response to an external stimulus needs further research.

We did observe higher baseline cortisol levels in unresolved/disorganized fathers compared to fathers with an organized AR. This finding is in line with previous studies demonstrating that disorganized children and adults with an unresolved AR displayed higher levels of cortisol levels compared to organized individuals [39]. Prior research in healthy male students showed increased baseline (non-stressed) cortisol levels in the afternoon, but not at other time points during the day, in insecurely compared to securely attached males. Additionally, associations between insecurity and higher cortisol values following interpersonal challenges have been observed [40]. Former studies including only female participants (children, adolescents and mothers) indicated a flatter cortisol awakening response in (anxiously) insecurely attached participants [41,42], but no differences in basal cortisol concentrations several hours after awakening and during the course of the day were observed [42].

Here, cortisol levels tended to decrease in response to the AAP; however, this result did not reach statistical significance. Krause et al. [11] reported a significant decrease in cortisol after AAP administrations in lactating postpartum women. In our pilot study decreases in cortisol were paralleled by increases in oxytocin concentrations. Although there was no significant relationship between percent changes in oxytocin and cortisol, this result is in line with earlier findings indicating that the release of oxytocin buffers the activity of the neuroendocrine stress system [17,18,19]. Higher cortisol reactivity in response to the AAI was observed in patients with IST and dismissing attachment compared to patients with IST and secure attachment and subjects of the control group with either secure or dismissing attachment [3]. Differences in cortisol reactivity between AR and health conditions might be explained by variations in the activation of the HPA-axis or brain areas involved in stress processing, such as the amygdala, affecting the neuroendocrine stress system (for an overview see [10]). We, however, found no differences in cortisol reactivity regarding AR, which could be due to the relatively small sample size of our study or the character of our study population (medically healthy and mentally stable men who recently became a father). In addition, it is worth mentioning that prior research involving HPA-axis reactivity in response to a laboratory stressor, such as the TSST or the cold pressor test, in the context of AR also reported inconsistent findings [4,40,43,44]. Future studies with larger sample sizes for more robust observations have to investigate the role of possible covariates and laboratory conditions that might influence the biological outcome variables.

This is the first study to co-investigate DHEA concentrations, a relevant biomarker of acute stress [23], in the context of AAP stimulation. DHEA has beneficial psychological effects during acute stress and has a protective role during the stress response by antagonizing the effects of cortisol [22,23]. We observed no significant changes in DHEA concentration in response to AAP administration and no differences in baseline and reactivity measures of DHEA between different AR groups, which could indicate that DHEA concentrations—at least in saliva—are not directly related to AR and/or is not responsive to attachment-related stimuli. Again, the comparability between DHEA levels in saliva and blood and their reactivity in response to an external stimulus could be further explored by future research.

## 5. Limitations and Future Directions

Limitations of our pilot study are mainly related to the cross-sectional design and the relatively modest sample size, which particularly limited detailed investigations of the differential effects of the attachment subgroups. The relatively low number of participants also demonstrates that the recruitment of men who recently became fathers is much more challenging compared to mothers who recently gave birth to their children as all men who volunteered and provided written informed consent for study participation were also included in this investigation. Future studies should consider the relatively low interest rate of men observed in our study when planning future studies and cohort sizes. A larger sample size would also allow the robust inclusion of possible confounders/covariates, such as age, smoking and baseline biomarker concentrations. In addition, further research should focus on determining the influence of psychological and clinical symptomology and psychological factors of risk and resilience in analogy to the observations made on mothers (e.g., psychosocial stress burden and social support) in the analyses [11]. Based on the AAP effects observed in mothers [11], we conducted a power analysis to estimate the number of necessary cases to find significant between-group differences. Noteworthily, the power analysis for the between-group comparison depends on the expected distribution of the dichotomous scaled variables. In the power analysis, we hypothesized the distributions of the certain –uncertain and organized–disorganized possible relative frequencies of the smaller group: (a) 40 %, (b) 30 % and (c) 20 %, respectively. With N = 40, α = 0.05 and 1 − β = 0.80, the confirmation of the alternative hypothesis can be expected if effect sizes d exceed the following limits: in case (a), 0.82; in case (b), 0.88; and in case (c), 1.01. Somewhat moderate effect sizes are sufficient to reach the tendency limit: in case (a), 0.70; in case (b), 0.75; and in case (c), 0.85. Effect sizes *d* around 0.80 are defined as “strong”, and the value is also considered as the limit for the proof of the effectiveness of a psychotherapeutic procedure, method or technique. As a result, a total cohort size of N = 40 was targeted, but only N = 26 male individuals could be recruited. Nevertheless, these calculations and numbers need empirical proof, and confounders have to be tested for their influences on the strength of between-group differences. Future studies have to confirm and demonstrate the robustness of our initial finding.

Another methodological limitation is related to the bioanalytical measurement of oxytocin in saliva which is controversially discussed in the literature. Assessing salivary oxytocin by immunoassay has been considered problematic because of its fast degradation, molecular weight and impurity [45], while later studies (e.g., [28,46,47,48,49]) described the assessment of oxytocin in saliva as a reliable and valid method. An additional important aspect to be considered is related to the stability of the reported AR; recent fatherhood could have influenced the attachment status of these men, potentially introducing bias into our study and limiting the validity of our findings to men in general. Finally, the AR of both mothers and fathers should be—at best—co-assessed in future studies. This approach would allow drawing a more detailed picture of the intergenerational transmission of AR. Finally, it is important to note that a clinical evaluation of the biomarker levels and their use in any interventional settings also requires future investigation.

## 6. Conclusions

The results of this pilot study imply that the AAP is not only a valid instrument for assessing attachment status but can also be used as an instrument to psychobiologically stimulate the attachment system in both women and men of young parenthood. Furthermore, our findings in mentally stable fathers indicate underlying changes in the functioning of the oxytocinergic and neuroendocrine system with AR and may be important for future investigations in psychobiological stress research and intervention studies.

## Figures and Tables

**Table 1:** Sociodemographic, clinical characteristics and salivary biomarker results of the participants for the total cohort and separately for fathers with an organized and disorganized attachment representation according to the AAP classification. Differences between the organized and the disorganized/unresolved group were calculated using independent *t*-test (normally distributed data), Mann–Whitney U test (non-normally distributed data), or χ^2^-test (binary variables).

	Total	Organized	Unresolved/ Disorganized	*t*-Test/ MWU-Test/ χ^2^-Test
Sociodemographic and Clinical Characteristics	N = 26	*n* = 21	*n* = 5	
Age in years, M (SD)	33 (5.2)	31.86 (4.14)	38 (6.63)	t_24_ = −2.66, *p* = 0.014
Academic education, *n*, yes (%)	15 (57.7)	12 (57.1)	3 (60)	χ^2^ = 0.01, *p* = 0.91
Family income < 4000 € per month (self-report), *n* (%)	11 (42)	10 (47.6)	1 (20)	χ^2^ = 1.26, *p* = 0.26
Cigarette smoking ^a^, yes, *n* (%)	7 (26.9)	3 (14.3)	4 (80)	χ^2^ = 7.9, *p* = 0.005
BMI, kg/m^2^, M (SD)	26.7 (5.4)	26.93 (5.84)	25.54 (2.72)	U = 52, z = −0.03, *p* = 0.97
Migration background, yes, *n* (%)	5 (19.2)	3 (14.3)	2 (40)	χ^2^ = 1.72, *p* = 0.19
Number of children, M (SD)	1.7 (0.56)	1.61 (0.59)	1.8 (0.45)	t_24_ = −0.64, *p* = 0.53
Lifetime psychiatric diagnosis, yes, *n* (%)	3 (1.5)	2 (9.5)	1 (20)	χ^2^ = 0.43, *p* = 0.51
Duration AAP (min), M (SD)	17.64 (5.42)	17.25 (5.29)	19.26 (6.3)	t_24_ = −0.74, *p* = 0.47
Attachment representation, *n* (%)				
Secure (F)	5 (19.2)	NA	NA	
Preoccupied (E)	7 (26.9)	NA	NA	
Dismissing (D)	9 (34.6)	NA	NA	
Unresolved (U)	5 (19.2)	NA	NA	
**Biological Markers**				
Oxytcin t_0_, 1 M (SD)	0.70 (0.38)	0.73 (0.39)	0.58 (0.32)	t_24_ = 0.82, *p* = 0.42
Oxytocin t_1_, M (SD)	1.15 (1.14)	1.06 (1.02)	1.54 (1.67)	U = 41, z = −0.75, *p* = 0.49
Cortisol t_0_, M (SD)	0.28 (0.21)	0.24 (0.18)	0.47 (0.23)	t_24_ = −2.48, *p* = 0.021
Cortisol t_1_, M (SD)	0.24 (0.17)	0.22 (0.17)	0.34 (0.18)	t_24_ = −1.4, *p* = 0.17
DHEA t_0_^b^, M (SD)	368.76 (202.73)	377.46 (220.51)	333.92 (117.21)	t_23_ = 0.42, *p* = 0.68
DHEA t_1_^c^, M (SD)	391.64 (231.22)	381.45 (197.36)	428.34 (355.45)	t_21_ = 0.39, *p* = 0.70
Oxytocin response %, M (SD)	84.06 (183.52)	72.76 (197.41)	131.51 (110.27)	U = 84, z = −2, *p* = 0.041
Cortisol response %, M (SD)	10.23 (66.17)	16.80 (70.63)	−17.32 (35.26)	U = 36, z = −1.1, *p* = 0.31
DHEA response % ^c^, M (SD)	62.77 (289.01)	70.75 (325.51)	34.06 (88.18)	U = 51, z = 0.45, *p* = 0.69

^a^ Data available from *n* = 24 participants; ^b^ *n* = 25 participants; ^c^ *n* = 23 participants. AAP, Adult Attachment Projective Picture System; DHEA, dehydroepiandrosterone; MWU, Mann–Whitney U test; NA, not applicable; SD, standard deviation; *t*-test, independent students *t*-test; χ^2^-test, Chi-square-test.

## Data Availability

Data will be made available upon request.
